# Maturation of suprathreshold auditory nerve activity involves cochlear CGRP–receptor complex formation

**DOI:** 10.14814/phy2.12869

**Published:** 2016-07-20

**Authors:** Ian M. Dickerson, Rhiannon Bussey‐Gaborski, Joseph C. Holt, Paivi M. Jordan, Anne E. Luebke

**Affiliations:** ^1^ Deptartment of NeuroscienceUniversity of Rochester Medical CenterRochesterNew York; ^2^ Deptartment of Biomedical EngineeringUniversity of Rochester Medical CenterRochesterNew York; ^3^ Deptartment of OtolaryngologyUniversity of Rochester Medical CenterRochesterNew York

**Keywords:** CGRP, CGRP‐RCP, CLR, cochlea, cochlear nerve, co‐immunoprecipitation, developmental, efferent, juvenile, lateral olivocochlear efferents, mouse, RAMP1, sensory coding

## Abstract

In adult animals, the neuropeptide calcitonin gene‐related peptide (CGRP) is contained in cochlear efferent fibers projecting out to the cochlea, and contributes to increased suprathreshold sound‐evoked activity in the adult auditory nerve. Similarly, CGRP applied to the lateral‐line organ (hair cell organ) increases afferent nerve activity in adult frogs (post‐metamorphic day 30), yet this increase is developmentally delayed from post‐metamorphic day 4–30. In this study, we discovered that there was also a developmental delay in increased suprathreshold sound‐evoked activity auditory nerve between juvenile and adult mice similar to what had been observed previously in frog. Moreover, juvenile mice with a targeted deletion of the *α* CGRP gene [CGRP null (−/−)] did not show a similar developmental increase in nerve activity, suggesting CGRP signaling is involved. This developmental delay is not due to a delay in CGRP expression, but instead is due to a delay in receptor formation. We observed that the increase in sound‐evoked nerve activity is correlated with increased formation of cochlear CGRP receptors, which require three complexed proteins (CLR, RAMP1, RCP) to be functional. CGRP receptor formation in the cochlea was incomplete at 1 month of age (juvenile), but complete by 3 months (adult), which corresponded to the onset of suprathreshold enhancement of sound‐evoked activity in wild‐type animals. Taken together, these data support a model for cochlear function that is enhanced by maturation of CGRP receptor complexes.

## Introduction

All mammalian hair cell systems contain an efferent innervation. Calcitonin gene‐related peptide (CGRP) is a 37‐amino acid neuropeptide contained in cochlear efferent neurons that innervate mammalian and amphibian hair cells (Simmons 2002). We discovered previously that the loss of the efferent neurotransmitter CGRP decreased sound‐evoked activity in the cochlear nerve of mature mice (Maison et al. 2003b). In frog, CGRP causes a similar increase in nerve activity when applied to the lateral line hair cell organ. Interestingly, this increased nerve activity is detected in adult frogs, post‐metamorphic (PM) day 30, yet is not present in juvenile frogs, PM day 4 (Bailey & Sewell, 2000b). We wondered if a similar maturation of CGRP responsiveness is present in the mouse cochlea.

Since CGRP expression does not differ between juvenile and adult mice (Maison et al. 2003a,b), we focused our attention on the receptor for CGRP, which is a G‐protein coupled receptor (GPCR) composed of three proteins: calcitonin‐like receptor (CLR), a 7‐transmembrane GPCR; receptor activity‐modifying protein (RAMP1), a single transmembrane protein that contributes pharmacologic specificity to CLR and aids in trafficking CLR to the cell surface; and CGRP‐receptor component protein (RCP), an intracellular peripheral membrane protein that enables signaling from the CLR/RAMP1 heterodimer (McLatchie et al. 1998; Evans et al. 2000; Egea and Dickerson 2012). The three proteins that constitute a functional CGRP receptor (CLR, RAMP1, RCP) co‐immunoprecipitate from cell lysates, and this interaction represents formation of a functional receptor complex (Evans et al. 2000; Prado et al. 2001). Importantly, direct interaction between RCP and CLR is required for signaling at CLR, and this interaction can be detected by co‐immunoprecipitation (Egea and Dickerson 2012). Thus, co‐immunoprecipitation of CLR with RCP can be used as an indicator of CGRP receptor competency, and is a predictor of CGRP efficacy.

To investigate efficacy of CGRP signaling during maturation of the murine cochlea, we examined transgenic mice with a targeted deletion of the *α*CGRP gene as juveniles and adults. First, using distortion‐product otoacoustic emissions (DPOAEs), and auditory brainstem recordings (ABRs), we compared the outer hair cell function and cochlear nerve activity between CGRP (−/−) null and CGRP (+/+) wild‐type adult and juvenile mice. Secondly, we monitored expression and interaction of CLR and RCP in juvenile and adult mice (CGRP (+/+) and correlated receptor function with cochlear function.

Our data from these studies supports a model where interaction between receptor proteins regulates CGRP efficacy in the cochlea, and this receptor complex formation changes during development in parallel with the efficacy of CGRP signaling.

## Methods

### Animals

*α*CGRP null (−/−) and wild‐type (+/+) transgenic mouse lines were created on a pure 129SvEv background, and characterized by the Emeson laboratory, shipped to the Luebke laboratory, maintained as heterozygotes, and genotyped using methods described by Lu et al. (1999). Mice, of either sex, have a targeted deletion of *α*CGRP, produced by tissue‐specific alternative splicing of the calcitonin/*α*CGRP gene while leaving the highly homologous *β*‐CGRP gene intact (Lu et al., 1999). The loss of *α*CGRP in the vasculature of null animals was compensated by *β*CGRP, and was not associated with abnormalities in heart rate or blood pressure under basal or exercise‐induced conditions, which could have been confounding issues in our study. The 129SvEv CGRP (+/−) mice were paired to generate homozygous CGRP (−/−) and CGRP (+/+) offspring used for all mouse studies. Equal numbers of male and female mice were used for all experiments. Mice were euthanized with an intraperitoneal injection of pentobarbital (50 mg/kg) and the cochlear and brain tissues retrieved. When tissues were used for immunohistochemistry, the anesthetized mice were euthanized by an intracardiac perfusion with PBS followed by 4% paraformaldehyde in PBS. The care and use of these animals were approved by the University of Rochester's Institutional Animal Care and Use Committee.

### Distortion‐product otoacoustic emission response measurements

Mice were sedated with acepromazine (10 mg/kg i.p.) and ketamine (100 mg/kg i.p.). DPOAEs at 2*f*_1_−*f*_2_ were recorded with an acoustic assembly consisting of two high frequency speakers (Tucker Davis EC1) to generate primary tones (*f*_1_ and *f*_2_ with *f*_2_/*f*_1_ = 1.2 and f_2_ level 10 dB < *f*_1_ level) and a ER10B^+^ microphone to record ear‐canal sound pressures. Stimuli were generated digitally, and ear‐canal sound pressure was amplified and digitally sampled, FFTs were computed and averaged over 5 consecutive waveform traces, and 2*f*_1_−*f*_2_ DPOAE amplitude and surrounding noise floor were extracted. SmartEP (Intelligent Hearing Systems, Miami, FL) was used for data acquisition and collection.

### Auditory Brainstem responses measurements

Animals were sedated as for DPOAE testing. Needle electrodes were inserted at the vertex and pinna, with a ground near the tail. ABR potentials were evoked with 5‐msec tone pips (0.5‐msec rise‐fall with a cos^2^ onset envelope, delivered at a rate of 35/sec). The response was amplified (10,000 X), filtered (100–3 kHz), and averaged with an analog‐digital (AD) board using Smart EP data‐acquisition system (Intelligent Hearing systems, Miami, FL) delivered through Tucker Davis EC1 speakers controlled with the Tucker Davis ED‐1 speaker driver. Sound levels were raised in 5–10 dB steps from 10 dB below threshold up to 80 dB SPL. At each sound level, 512 responses were averaged (with stimulus polarity alternated), using an ‘artifact reject’ whereby response waveforms were discarded when peak‐to‐peak amplitude exceeded 15 mV. Upon visual inspection of stacked waveforms, ‘threshold’ was defined as the lowest SPL level at which any wave could be detected, usually corresponding to the level step just below that at which the peak‐peak response amplitude rose significantly above the noise floor (~0.25 mV). For amplitude vs. level functions, the wave I peak was identified by visual inspection at each sound level and the peak‐to‐peak amplitude was computed using the Smart EP software (Intelligent Hearing Systems, Miami, FL).

### Antibodies

ChAT (Millipore, Billerica, MA; AB144P lot JC1618187, 1:100 dilution or lot NG1780580, 1:250–1:500 dilution) antibodies, generated against the human placental enzyme, were used to label efferent innervation in the cochlea. CGRP (MU33) antibody (1:500 dilution) was generated against the amidated carboxyl 7 amino acids of rat *α*CGRP (Rosenblatt and Dickerson, 1997). NY1024 is an anti‐CLR rabbit polyclonal antibody raised against the peptide CWNDVAAGTESMQ, 1065 is a chicken anti‐RCP antibody raised against the peptide EEQIEALLHTVT, NY1047 is an anti‐RCP rabbit polyclonal antibody raised against the peptide GPEDEQSKSTSND, and a polyclonal antibody against human myosin VIIa (Myo7A, Proteus Biosciences Inc., #25–6790, 10 *μ*g/m). (Prado et al. 2001; Ma et al. 2003; Glaser et al. 2007).

### Morphological/Immunohistochemical (IHC) Studies

Mice were anesthetized with ketamine (80 mg/kg)/xylazine (5 mg/kg) and perfused with heparinized PBS followed by 4% paraformaldehyde (PFA). Animals were then decapitated with head postfixed in 4% PFA overnight at 4°C. Cochleae were subsequently dissected and were blocked with 5% normal donkey sera (Jackson Immunoresearch, Westgrove PA) and 0.5% Triton X in 0.1 mol/L‐phosphate buffer (PB), and then incubated with primary antibodies (see below) in PB overnight. Following washing, cochlear tissues were reacted with the appropriate Alexa Fluor^®^‐conjugated secondary antibodies (Molecular Probes/Invitrogen, Grand Island, NY;) at 1:500 in PB for 2–3 h. The cochlear tissues were then washed in PB, stained w/DAPI (10 *μ*g/mL, Sigma, St. Louis, MO), and mounted.

### Imaging/Quantification

For visualization of labeled cochleae, an Olympus FV1000 laser scanning confocal microscope (URSMD Light Microscopy Core) with a PLAPON 60× oil objective and sequential scanning option was used to capture images of labeled structures in the linear range. Maximum intensity projections were created using FV1000 software, and Adobe Photoshop and Illustrator were used to compile figures.

### Preparation of tissue lysates

Tissue lysates were prepared by homogenizing cochleae at high speed in a Brinkmann Polytron for 15 sec in PTN50 buffer (50 mmol/L sodium phosphate, pH 7.4, 1% Triton X‐100, 50 mmol/L NaCl) containing protease inhibitors (50 *μ*g/mL Lima Bean Trypsin Inhibitor, 2 *μ*g/mL Leupeptin, 16 *μ*g/mL Benzamidine, 2 *μ*g/mL Pepstatin A, 300 *μ*g/mL phenylmethylsulfonyl fluoride) (Evans et al. 2000) and incubating on ice for 30 min. Cell debris was pelleted by centrifugation for 10 min @ 700 xg, and the supernate (lysate) was saved for analysis. For cochlear lysates, proteins were normalized by cochlea, as cochleae were ground with epithelium, ganglion cells, and bone all within the bony labyrinth, so equal volumes of cochlear lysate (approximately 1/6 cochlea/lane for Western blotting). Protein concentrations of tissue culture cell lysates were determined, using the Micro BCA Assay (Pierce, Rockford, IL).

### Immunoprecipitations

RCP was immunoprecipitated as published (Egea and Dickerson 2012) from lysate from four cochleae overnight at 4^o^ using chicken polyclonal antibody against RCP (1065) (Prado et al. 2001; Ma et al. 2003). The immune complexes were captured using immobilized anti‐chicken IgY (Promega, Madison, WI). The immunoprecipitates was boiled in SDS‐Laemmli sample buffer, and analyzed by Western blot, using rabbit polyclonal antibody against CLR (NY1024).

### Western Blot analysis

Immunoprecipitates and extracts were resolved by SDS‐PAGE on 8–15% gels and transferred to polyvinylidene difluoride membrane (Bio‐Rad, Hercules, CA), and incubated with antibodies directed against RCP (1065 diluted 1:10,000; NY1047 diluted 1:6,000) or CLR (NY 1024 diluted 1:10,000) or myosin 7a, diluted 1:10,000. Membranes were incubated with antibodies for 1–3 h at room temperature in phosphate‐buffered saline/0.04% Tween (PBS‐T) plus 1% non‐fat milk. Membranes were then washed three times with PBS‐T/milk and incubated with a 1:50,000 dilution of either donkey anti‐chicken (Jackson Immunoresearch) or donkey anti‐rabbit (Amersham Pharmacia Biotech) secondary antibodies conjugated to horseradish peroxidase for 60 min. Membranes were then washed three times with PBS‐T, incubated in SuperSignal West Dura Extended Chemiluminescent Substrate (Pierce) for 5 min, and exposed to X‐ray film (Kodak XAR).

## Results

**Figure 1:**
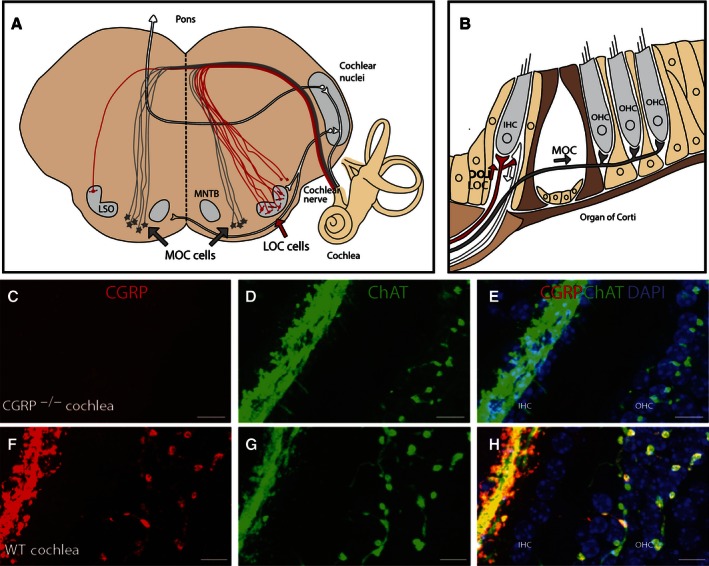
Loss of *α* CGRP does not alter the cholinergic olivocochlear (OC) projection to the cochlea. (A) Schematic of midbrain/brainstem showing cochlear efferent fibers origination [medial (MOC) lateral (LOC) olivocochlear fibers]. (B) Schematic of cochlear endorgan showing location of afferent (white) and efferent (black‐MOC & red‐LOC) fiber projections. CGRP is present in both LOC and MOC efferent fibers. (C) CGRP is absent in the neuroepithelium of *α* CGRP(−/−) mouse cochlea, but choline acetyl transferase (ChAT) expression is unaffected (D, E). (F–H) CGRP is abundantly present in fibers ending on outer hair cells (MOC) and afferents (LOC) of CGRP(+/+) mouse cochlea with a distribution overlapping ChAT expression (G & H) as illustrated in the composite images (H). Nuclei are labeled blue, staining for CGRP and ChAT are in red and green, with overlap indicated in yellow and orange. Scale bars=10 *μ*m. CGRP, calcitonin gene‐related peptide.

A light microscopic examination revealed no gross structural abnormalities in the cochlea of *α*CGRP(−/−) null animals, with regard to hair cell location and number and afferent innervation. Dissected cochleae from *α*CGRP(+/+) and (−/−) animals were immunostained for CGRP and choline acetyltransferase (ChAT) to visualize cholinergic efferent innervation and was visualized, using confocal microscopy (Fig. 1). Consistent with cochlear efferent innervation ChAT‐positive fibers and associated varicosities were identified in the cochlea in close proximity to the base of outer hair cells due primarily to medial olivocochlear efferent fibers (MOC‐Fig.1A and B) and cochlear afferents due to lateral (OC) efferent fibers (Fig. 1A and B). In CGRP(+/+) mice, there was an extensive overlap in the distributions of CGRP and ChAT immunoreactive fibers and varicosities (Fig. 1F and G). The overlap can be further appreciated in Figure 1H, where immunoreactive varicosities positive for both ChAT and CGRP appear as yellow and orange. Immunohistochemistry in *α*CGRP(−/−) mice revealed that CGRP immunoreactive fibers and varicosities were absent in the cochlea (Fig. 1C). When images from CGRP and ChAT immunostaining were merged, only ChAT+ fibers and varicosities were present (Fig. 1E). ChAT labeling, however, appeared similar between CGRP(+/+) and CGRP(−/−) mice (Fig.1D and G).

### Cochlear function maturation:

**Figure 2:**
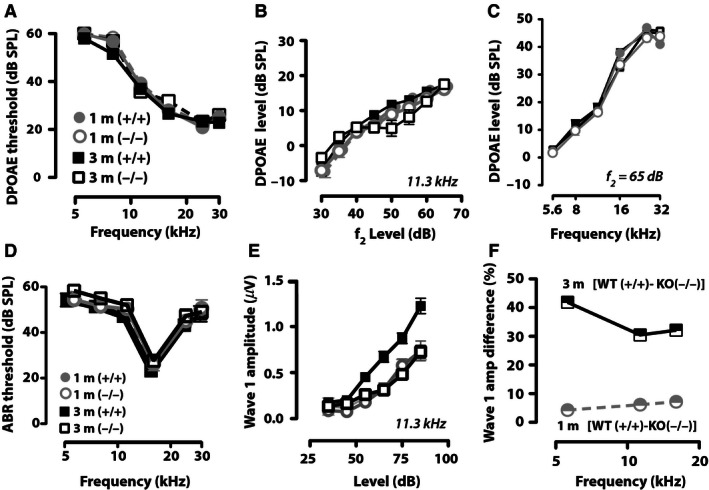
Cochlear ABR suprathreshold measures increase during juvenile to adult maturation. DPOAE thresholds (A) did not differ significantly between CGRP (+/+) or (−/−) animals at either 1 month (1 m) or 3 months (3 m), nor did (B) DPOAE growth functions differ (growth of DPOAE amplitude with sound level at all frequencies tested), shown here for 11.3 kHz) nor did (C) DPOAE output levels to 75/65 dB primaries did not differ between CGRP (+/+) and (−/−) animals. (D–F) ABR thresholds (D) did not differ between CGRP (+/+) and (−/−) animals (D) at either 1 month (1 m) or 3 months (3 m). (E) In contrast to DPOAE growth functions, ABR growth functions did differ between CGRP (+/+) and (−/−) animals at 3 m, but not when animals were tested at 1 m. (F) The percent difference in suprathreshold ABR wave 1 amplitude between CGRP (+/+) and (−/−) animals at 3 m (half‐colored black squares) was between 25–45% for frequencies between 5.4–16 kHz. Whereas there were no significant differences between CGRP (+/+) and CGRP (−/−) animals at 1 m. The mean group values and SEM. are shown, and each group contained 15 mice. ABR, auditory brainstem recordings; CGRP, calcitonin gene‐related peptide; DPOAE, distortion‐product otoacoustic emissions.

To investigate the effect maturation has on cochlear outer hair cell function, we measured DPOAEs in both CGRP (+/+) and CGRP (−/−) mice at juvenile (1 month) and adult (3 month) ages. Maturation had no significant effect on DPOAE thresholds (Fig. 2A), or DPOAE growth functions as shown in Figure 2B for *f*_2_ = 11.3 kHz, or suprathreshold responses of DPOAE amplitudes (*f*_2_ = 5.6–30 kHz; Fig. 2C). Loss of CGRP also had no significant effect on DPOAE thresholds (Fig. 2A), or DPOAE growth functions (Fig. 2B) or DPOAE amplitudes (Fig 2C). There were also no significant differences in ABR thresholds between juvenile and adult CGRP (+/+) mice animals or ABR threshold differences in mice with (+/+) or without CGRP (−/−) as shown in Figure 2D.

However, in adult animals, there were differences in suprathreshold cochlear nerve activity (as assessed by the amplitude of wave 1 of the ABR) between CGRP (+/+) and CGRP (−/−) mice (Fig. 2E). Interestingly, this difference in cochlear nerve activity between CGRP (+/+) and CGRP (−/−) was not detected in juvenile animals, suggesting that the influence of CGRP on suprathreshold cochlear responses is only present in adult animals. The difference in suprathreshold responses for juvenile (light gray circles) and adult (black squares) wild‐type animals (+/+) are shown in Figure 2E for wave 1 amplitudes to 11.3 kHz. When the differences in suprathreshold responses are plotted between CGRP (+/+) and (−/−) animals, for all frequencies assessed, there were negligible CGRP‐induced enhancements of suprathreshold cochlear responses in juvenile animals (difference between CGRP (+/+) CGRP (−/−) response; (Fig. 2F, half‐gray circles), but a 30–40% enhancement in suprathreshold responses was observed in adult CGRP (+/+) animals (half black squares).

### Maturation of CGRP receptor complex

**Figure 3:**
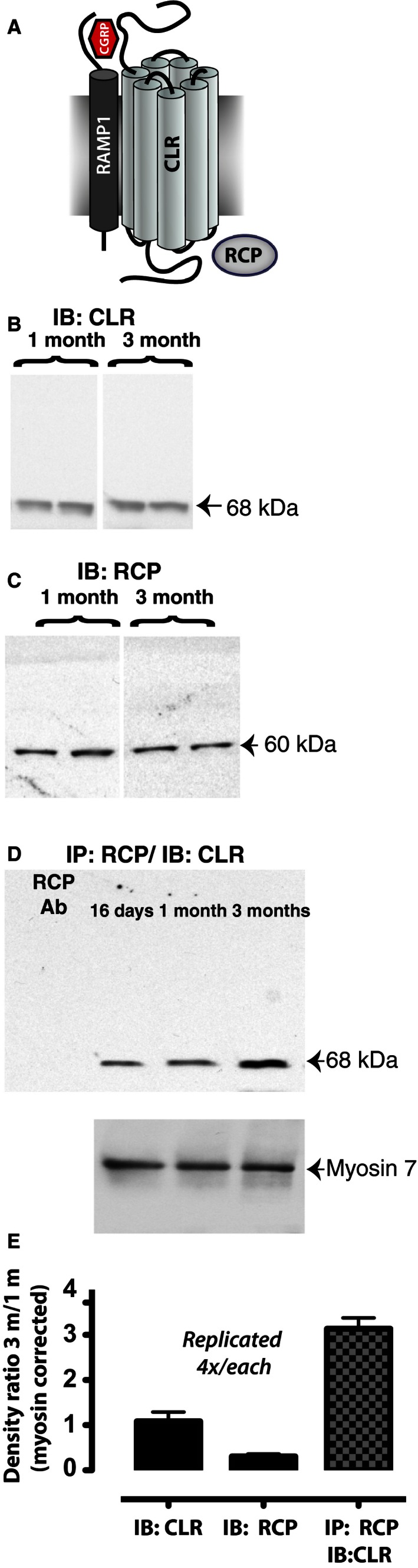
CGRP efficacy correlates with receptor complex formation during juvenile to adult maturation. (A) Model for the CGRP receptor complex, composed of transmembrane proteins CLR and RAMP1, and intracellular peripheral membrane protein RCP. (B) Western blot of mouse cochlea for CLR expression at 1 and 3 months. (C) Western blot for RCP at 1 and 3 months. (D) CLR‐RCP co‐immunoprecipitation. Cochlear lysate was immunoprecipitated with a chicken anti‐RCP polyclonal antibody, and the immunoprecipitate analyzed by Western blot with a rabbit anti‐CLR polyclonal antibody. (E) Quantification of RCP‐CLR immunoblot and co‐immunoprecipitation studies. No change between individual components of the CGRP receptor between 1 m and 3 m of age, yet threefold more CGRP receptor co‐immunoprecipitated at 3 m when compared to 1 m. CGRP, calcitonin gene‐related peptide; CLR, calcitonin‐like receptor; RCP, receptor component protein.

The increased sensitivity to suprathreshold auditory responses that we observed between the first and third month suggests a change in cochlear efferent function. The fact that this increase in function was not observed in CGRP (−/−) null mice suggested a role for CGRP signaling in cochlear maturation. A delay in CGRP signaling could be due to either a delay in CGRP expression or a delay in receptor signaling. We and others have not found a difference in CGRP expression in the juvenile to adult cochlea (Maison et al. 2003a,b; so we therefore looked for expression of the CGRP receptor proteins CLR and RCP (Fig. 3A) at 1 month and 3 months of development. We did not investigate CGRP receptor protein expression in CGRP (−/−) null mice since their CGRP signaling was eliminated by the loss of the CGRP peptide. However, in wild‐type mice, CLR was detected by Western blot at the predicted 68 kDa size indicative of mature processed receptor (Evans et al. 2000; Prado et al. 2001; Glaser et al. 2007), but no change in cochlear CLR expression was detected between 1 month and 3 months in the mouse (Fig. 3B). Similarly, no increase was observed in expression of cochlear RCP between one and 3 months (Fig. 3C). However, a change in RCP–CLR interaction was detected during development. RCP was immunoprecipitated from cochlear lysates prepared from 1 month and 3 month old mice, the same mice used in Figure 2 studies where cochlear function was confirmed. This RCP immunoprecipitate was analyzed by Western blot for CLR (Fig. 3D). We observed an increase in CLR that co‐immunoprecipitated with RCP at 3 months compared to 1 month or postnatal day 16 (P16), even though the total amount of RCP decreased slightly at 3 months (Fig. 3D and E). Thus, the interaction between CLR and RCP is strengthened at 3 months, and this increased cochlear RCP–CLR interaction parallels increased auditory nerve activity.

## Discussion

In this study, we established that mice develop a significant increase in auditory nerve activity to suprathreshold sounds between 1 month (juvenile) and 1 months of age (adult). Juvenile mice with a targeted deletion of the *α*CGRP gene [CGRP null (−/−)] did not show a similar developmental increase in nerve activity, suggesting CGRP signaling is involved. Immunohistochemistry confirmed that CGRP was absent in the cochlea of CGRP null (−/−) mice, while ChAT staining suggested the gross development of the olivocochlear efferent system was unaltered. In agreement with a role for CGRP signaling in maturation, formation of functional CGRP receptors in the cochlea was associated with increases in cochlear nerve activity.

While our current study focused on cochlear maturation, there are parallels with other hair cell systems, as, CGRP is a conserved neuropeptide in many hair cell efferent feedback systems (Simmons 2002). A similar age‐related increase in CGRP‐responsiveness has been observed in the frog's (*Xenopus laevis)* lateral‐line organ, where CGRP‐responsiveness of hair cells was first observed at post‐metamorphic day 6 and then progressively increased, reaching a maximum by post‐metamorphic day 30 (Bailey and Sewell 2000a). This delay in CGRP responsiveness in *Xenopus* was also not due to the absence of CGRP in frog lateral‐line efferent fibers at post‐metamorphic day 6 (Bailey and Sewell 2000a), so presumably it also involves CGRP receptor formation. In the vestibular system, there is an increase in the gain of the vestibular ocular reflex (VOR) from juvenile ages to adulthood (Faulstich et al. 2004). It is not known if this vestibular maturation also involves CGRP signaling maturation, yet CGRP signaling may also be involved, as we have recently determined that the loss of CGRP (−/−) in adult mice reduced the VOR gain by ~50% (Luebke et al. 2014).

While CGRP receptor maturation was not specifically studied, changes in auditory sensitivity with juvenile‐to‐adult maturation have been observed. Juvenile cochleae (kitten or mouse) are more vulnerable to damage from acoustic overexposures than are adult cochlea (Saunders and Chen 1982; Kujawa and Liberman 2006). For example, when 2‐month‐old kittens and adult cats were exposed to a 5 kHz pure tone at 105 dB, there was a permanent loss in cochlear microphonic sensitivities in the kittens, but not in the adult cats (Saunders and Chen 1982). Similarly, when juvenile and adult mice were exposed to the identical noise band, the juvenile mice exhibited a permanent threshold shift; whereas the adult mice were not affected (Kujawa and Liberman 2006). Sarro and Sanes found that juvenile gerbils displayed greater amplitude‐modulated (AM) detection thresholds than did adults (Sarro and Sanes 2010). Moreover, this difference from juvenile to adult was also evident in auditory cortical recordings, as juvenile animals showed immature depth sensitivities to sinusoidally AM‐modulated stimuli (Rosen et al. 2010). These data suggest that there are differences in noise susceptibility, cochlear microphonic activity and neural cortical activity in the auditory system between juvenile and adult animals, and juvenile‐to‐adult maturation of auditory signaling pathways (which may include CGRP) may play a role in these processes.

In summary, our data support a necessary role for CGRP signaling and receptor complex formation in juvenile‐to‐adult maturation of sound‐evoked cochlear nerve activity.

## Conflict of Interest

The authors declare no competing financial interests.
